# Genomic Epidemiology of Corynebacterium diphtheriae in New Caledonia

**DOI:** 10.1128/spectrum.04616-22

**Published:** 2023-04-12

**Authors:** Eve Tessier, Melanie Hennart, Edgar Badell, Virginie Passet, Julie Toubiana, Antoine Biron, Ann-Claire Gourinat, Audrey Merlet, Julien Colot, Sylvain Brisse

**Affiliations:** a CHU Nantes, Service de Bactériologie et des Contrôles Microbiologiques, Nantes, France; b Microbiology Laboratory, Centre Hospitalier Territorial Gaston Bourret, Nouméa, New Caledonia; c Institut Pasteur, Université Paris Cité, Biodiversity and Epidemiology of Bacterial Pathogens, Paris, France; d Sorbonne Université, Collège doctoral, Paris, France; e National Reference Center for the Corynebacteria of the diphtheriae complex, Paris, France; f Université Paris Cité, Department of General Pediatrics and Pediatric Infectious Diseases, Hôpital Necker–Enfants Malades, APHP, Paris, France; g Infectious diseases unit, Centre Hospitalier Territorial Gaston Bourret, Nouméa, New Caledonia; h Institut Pasteur de Nouvelle Calédonie, Groupe de Bactériologie médicale et environnementale Nouméa, New Caledonia; University of Pretoria

**Keywords:** *Corynebacterium diphtheriae*, diphtheria, New Caledonia, clinical presentation, genomic epidemiology, tropical island, whole-genome sequencing

## Abstract

An increasing number of isolations of Corynebacterium diphtheriae has been observed in recent years in the archipelago of New Caledonia. We aimed to analyze the clinical and microbiological features of samples with C. diphtheriae. All C. diphtheriae isolates identified in New Caledonia from May 2015 to May 2019 were included. For each case, a retrospective consultation of the patient files was conducted. Antimicrobial susceptibility phenotypes, *tox* gene and diphtheria toxin expression, biovar, and the genomic sequence were determined. Core genome multilocus sequence typing (cgMLST), 7-gene MLST, and search of genes of interest were performed from genomic assemblies. Fifty-eight isolates were included, with a median age of patients of 28 years (range: 9 days to 78 years). Cutaneous origin accounted for 51 of 58 (87.9%) isolates, and C. diphtheriae was associated with Staphylococcus aureus and/or Streptococcus pyogenes in three-quarters of cases. Half of cases came either from the main city Noumea (24%, 14/58) or from the sparsely populated island of Lifou (26%, 15/58). Six *tox*-positive isolates were identified, associated with recent travel to Vanuatu; 5 of these cases were linked and cgMLST confirmed recent transmission. Two cases of endocarditis in young female patients with a history of rheumatic fever involved *tox*-negative isolates. The 58 isolates were mostly susceptible to commonly used antibiotics. In particular, no isolate was resistant to the first-line molecules amoxicillin or erythromycin. Resistance to tetracycline was found in a genomic cluster of 17 (29%) isolates, 16 of which carried the *tetO* gene. There were 13 cgMLST sublineages, most of which were also observed in the neighboring country Australia. Cutaneous infections may harbor nontoxigenic C. diphtheriae isolates, which circulate largely silently in nonspecific wounds. The possible introduction of *tox*-positive strains from a neighboring island illustrates that diphtheria surveillance should be maintained in New Caledonia, and that immunization in neighboring islands must be improved. Genomic sequencing uncovers how genotypes circulate locally and across neighboring countries.

**IMPORTANCE** The analysis of C. diphtheriae from the tropical archipelago of New Caledonia revealed a high genetic diversity with sublineages that may be linked to Polynesia, Australia, or metropolitan France. Genomic typing allowed confirming or excluding suspected transmission events among cases and contacts. A highly prevalent tetracycline-resistant sublineage harboring the *tetO* gene was uncovered. Toxigenic isolates were observed from patients returning from Vanuatu, showing the importance of improving vaccination coverage in settings where it is insufficient. This study also illustrates the importance for diphtheria surveillance of the inclusion of isolates from cutaneous sources in addition to respiratory cases, in order to provide a more complete epidemiological picture of the diversity and transmission of C. diphtheriae.

## INTRODUCTION

Once a major cause of child mortality, diphtheria has been largely controlled following mass vaccination (1). However, 4,000 to 8,000 cases are reported each year worldwide (1, 2). Large recent outbreaks have been linked to insufficient local vaccination coverage rate following economic crises, political unrest and population displacements (3–5). Diphtheria is an infection caused mainly by C. diphtheriae, which is transmitted among humans. The disease has two main forms, pseudomembranous tonsillitis and cutaneous diphtheria. Other clinical presentations include systemic syndromes caused by the action of diphtheria toxin, endocarditis and secondary localizations due to hematogenous dissemination of nontoxigenic isolates (6, 7). Only some C. diphtheriae strains carry the *tox* gene, which codes for the diphtheria toxin. Although the bacterium typically transmits through droplets in respiratory infections, transmission can also occur through contact with infected or colonized skin lesions. Cutaneous diphtheria has become more frequent than the classical respiratory form in developed countries, and probably plays an important role during interepidemic periods in the tropics, where sporadic cases occur frequently.

The spatial distribution, local spread, and global dissemination of C. diphtheriae strains are largely undocumented. Genomic sequencing used in the context of epidemiological surveillance can help decipher the links between isolates and infer patterns of spread. So far, apart from outbreak situations (5, 8, 9), few genomic epidemiology studies of diphtheria have been performed (10–14).

New Caledonia, an overseas collectivity of France, is located in the South Pacific region; it is an island with tropical weather and a population of about 270, 000 people (15). This island system makes it an interesting case study to describe the diversity of C. diphtheriae isolates and their spread within the island and into or from it. Links with cases of diphtheria in neighboring countries such as Indonesia (16, 17) and Australia (18, 19) are unknown. Frequent travel between New Caledonia and mainland France may be accompanied by C. diphtheriae dissemination (12).

The aim of this study was to describe clinical, microbiological and genotypic characteristics of C. diphtheriae strains isolated in New Caledonia during 4 years. Clinical presentations, geographical localization and microbiological features of 58 cases of infection or carriage of C. diphtheriae were reported, and a genomic comparison of isolates from New Caledonia and other world regions was performed.

## RESULTS

### Demographic and clinical data.

**FIG 1:**
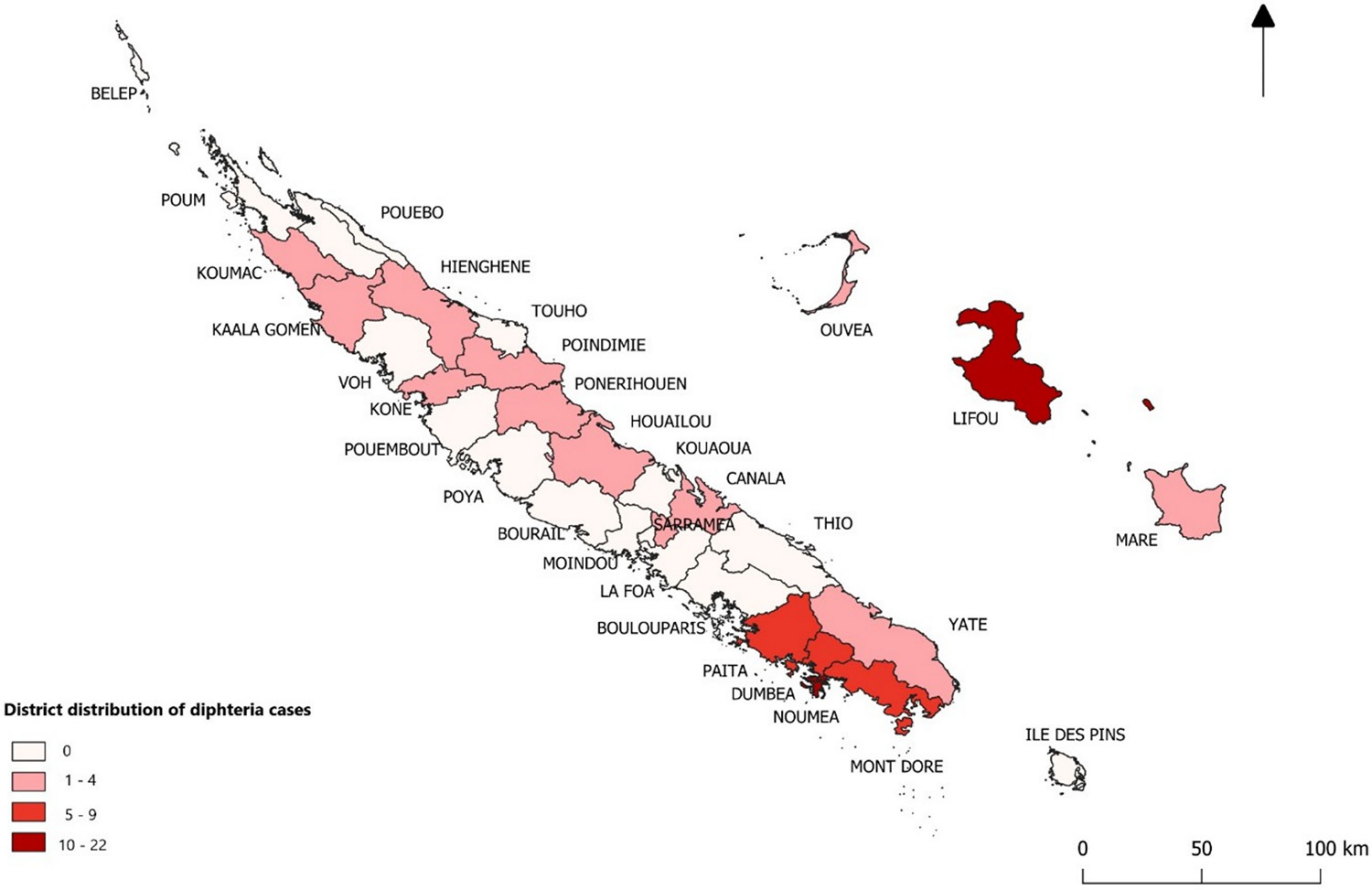
Geographic distribution of Corynebacterium diphtheriae cases in New Caledonia. The number of patients whose living place is in each district, is indicated as a heatmap (see key). The arrow indicates the direction of the North.

The demographic and clinical characteristics of the cases are presented in Table 1; 58 cases were included, with a sex ratio of 1.07 (30 men and 28 women). The median age of subjects was 28 years (range, 9 days to 78 years). The geographic distribution of cases (Fig. 1) was uneven; the two districts with most cases were Nouméa (14 cases, 24.1%) and Lifou island (15 cases, 25.9%).

**TABLE 1:** Characteristics of the isolates^*a*^

Study ID	ID in BIGSdb	Isolate ID	Sex	Yr	Province	Administrative district	Investigation group	Sample	Body location	Species	*Tox* gene PCR	Elek	ST (mLST)	Sublineage (internal ID = SL alias)	Genomic cluster	Biosample accession	Assembly accession
17	434	FRC0322	M	2015	North	Poindimié		Abscess	Lower limb	C. diphtheriae	Negative	NR	228	43 = SL228	278	ERS4330911	GCA_902808865.1
20	442	FRC0356	M	2015	South	Nouméa		Wound	Body trunk	C. diphtheriae	Negative	NR	228	43 = SL228	285	ERS4330918	GCA_902808925.1
22	140	FRC0411	F	2016	Islands	Lifou		Wound	Body trunk	C. diphtheriae	Negative	NR	416	82 = SL416	108	ERS4330931	GCA_902809175.1
24	478	FRC0456	F	2016	South	Yaté		Wound	Lower limb	C. diphtheriae	Negative	NR	228	43 = SL228	307	ERS4330949	GCA_902809315.1
25	484	FRC0469	M	2016	Islands	Maré		Wound	Lower limb	C. diphtheriae	Negative	NR	228	43 = SL228	312	ERS4330954	GCA_902809335.1
27	497	FRC0485	F	2017	South	Mont Dore (travel from Vanuatu)		Wound	Lower limb	C. diphtheriae	**Positive**	Positive	380	192 = SL380	321	ERS4330965	GCA_902809445.1
28	499	FRC0492	F	2017	Islands	Lifou	1	Wound	Lower limb	C. diphtheriae	Negative	NR	416	82 = SL416	108	ERS4330967	GCA_902809455.1
29	500	FRC0493	M	2017	Islands	Lifou	1	Wound	Lower limb	C. diphtheriae	Negative	NR	533	193 = SL533	322	ERS4330968	GCA_902809515.1
30	503	FRC0497	M	2017	Islands	Ouvéa		Wound	Lower limb	C. diphtheriae	Negative	NR	522	195 = SL522	324	ERS4330969	GCA_902809475.1
31	515	FRC0513	F	2017	South	Mont Dore		Abscess	Lower limb	C. diphtheriae	Negative	NR	228	43 = SL228	312	ERS4330974	GCA_902809525.1
35	523	FRC0524	F	2017	Islands	Lifou		Wound	Lower limb	C. diphtheriae	Negative	NR	416	82 = SL416	108	ERS4330979	GCA_902809585.1
36	527	FRC0533	M	2017	South	Nouméa		Wound	Upper limb	C. diphtheriae	Negative	NR	228	43 = SL228	342	ERS4330981	GCA_902809575.1
37	528	FRC0534	M	2017	South	Nouméa		Abscess	Lower limb	C. diphtheriae	Negative	NR	86	56 = SL86	75	ERS4330982	GCA_902809555.1
38	536	FRC0547	M	2017	South	Nouméa		Wound	Lower limb	C. diphtheriae	Negative	NR	524	45 = SL524	57	ERS4330989	GCA_902809715.1
40	1453	FRC0562	F	2017	North	Koné		Blood	Blood	C. diphtheriae	Negative	NR	228	43 = SL228	511	ERS13588169	GCA_947533805
39	1454	FRC0563	F	2017	South	Saraméa		Abscess	Upper limb	C. diphtheriae	Negative	NR	228	43 = SL228	702	ERS13588170	GCA_947533725
41	861	FRC0577	F	2017	South	Nouméa		Wound	Lower limb	C. diphtheriae	Negative	NR	228	43 = SL228	312	ERS13588127	GCA_947533785
43	862	FRC0578	M	2018	North	Ponerihouen		Abscess	Lower limb	C. diphtheriae	Negative	NR	232	50 = SL232	122	ERS13588128	GCA_947533555
44	863	FRC0579	F	2018	Islands	Lifou		Wound	Lower limb	C. diphtheriae	Negative	NR	605	48 = SL605	61	ERS13588129	GCA_947533575
42	864	FRC0580	F	2018	South	Nouméa		Wound	Lower limb	C. diphtheriae	Negative	NR	416	82 = SL416	108	ERS13588130	GCA_947533525
45	865	FRC0581	M	2018	Islands	Lifou		Abscess	Face	C. diphtheriae	Negative	NR	524	45 = SL524	57	ERS13588131	GCA_947533545
46	1426	FRC0597	F	2018	North	Hienghene		Abscess	Lower limb	C. diphtheriae	Negative	NR	228	43 = SL228	312	ERS13588165	GCA_947533775
47	1427	FRC0598	F	2018	South	Mont Dore		Abscess	Lower limb	C. diphtheriae	Negative	NR	228	43 = SL228	312	ERS13588166	GCA_947533815
48	868	FRC0599	F	2018	Islands	Maré		Wound	Lower limb	C. diphtheriae	Negative	NR	533	193 = SL533	322	ERS13588132	GCA_947533875
49	869	FRC0600	F	2018	South	Nouméa		Blood	Blood	C. diphtheriae	Negative	NR	416	82 = SL416	108	ERS13588133	GCA_947533645
50	1428	FRC0601	F	2018	South	Paita		Wound	Upper limb	C. diphtheriae	Negative	NR	228	43 = SL228	312	ERS13588167	GCA_947533795
51	870	FRC0602	F	2018	South	Paita		Wound	Face	C. diphtheriae	Negative	NR	228	43 = SL228	312	ERS13588134	GCA_947533515
52	1429	FRC0603	F	2018	South	Nouméa		Wound	Body trunk	C. diphtheriae	Negative	NR	228	43 = SL228	511	ERS13588168	GCA_947533715
60	962	FRC0713	M	2019	South	Dumbéa (travel from Vanuatu)	2	Wound	Upper limb	C. diphtheriae	**Positive**	Positive	120	227 = SL120	499	ERS13588135	GCA_947533615
61	963	FRC0714	M	2019	South	Dumbéa (travel from Vanuatu)	2	Wound	Upper limb	C. diphtheriae	**Positive**	Positive	120	227 = SL120	499	ERS13588136	GCA_947533915
63	964	FRC0715	M	2019	South	Dumbéa (travel from Vanuatu)	2	Wound	Upper limb	C. diphtheriae	**Positive**	Positive	120	227 = SL120	499	ERS13588137	GCA_947533535
64	965	FRC0716	F	2019	South	Dumbéa	2	Throat	Throat	C. diphtheriae	**Positive**	Positive	120	227 = SL120	499	ERS13588138	GCA_947533665
66	967	FRC0717	M	2019	South	Dumbéa	2	Wound	Lower limb	C. diphtheriae	**Positive**	Positive	120	227 = SL120	499	ERS13588139	GCA_947533565
74	968	FRC0718	M	2019	South	Nouméa		Abscess	Upper limb	C. diphtheriae	Negative	NR	228	43 = SL228	312	ERS13588140	GCA_947533885
59	969	FRC0719	F	2019	South	Nouméa		Blood	Blood	C. diphtheriae	Negative	NR	416	82 = SL416	108	ERS13588141	GCA_947533605
62	970	FRC0720	F	2019	Islands	Lifou		Wound	Lower limb	C. diphtheriae	Negative	NR	416	82 = SL416	108	ERS13588142	GCA_947533585
65	971	FRC0721	M	2019	South	Mont Dore		Abscess	Lower limb	C. diphtheriae	Negative	NR	228	43 = SL228	312	ERS13588143	GCA_947533765
67	972	FRC0722	M	2019	South	Nouméa		Abscess	Lower limb	C. diphtheriae	Negative	NR	416	82 = SL416	108	ERS13588144	GCA_947533655
68	973	FRC0723	M	2019	North	Canala		Wound	Lower limb	*C. belfantii*	Negative	NR	42	119 = SL226	500	ERS13588145	GCA_947533635
69	974	FRC0724	M	2019	Islands	Lifou		Wound	Lower limb	C. diphtheriae	Negative	NR	232	50 = SL232	122	ERS13588146	GCA_947533755
70	975	FRC0725	F	2019	Islands	Lifou		Wound	Lower limb	C. diphtheriae	Negative	NR	533	193 = SL533	322	ERS13588147	GCA_947533675
53	994	FRC0745	M	2018	South	Paita		Abscess	Lower limb	C. diphtheriae	Negative	NR	240	43 = SL228	232	ERS13588148	GCA_947533745
54	996	FRC0746	M	2018	South	Dumbéa		Wound	Lower limb	C. diphtheriae	Negative	NR	416	82 = SL416	108	ERS13588149	GCA_947533595
57	998	FRC0748	M	2018	South	Paita		Implantable chamber	Upper limb	C. diphtheriae	Negative	NR	687	42 = SL687	510	ERS13588150	GCA_947533625
58	999	FRC0749	F	2018	South	Nouméa		Wound	Lower limb	C. diphtheriae	Negative	NR	228	43 = SL228	511	ERS13588151	GCA_947533845
72	1000	FRC0750	M	2019	Islands	Lifou		Abscess	Upper limb	C. diphtheriae	Negative	NR	416	82 = SL416	108	ERS13588152	GCA_947533855
73	1001	FRC0751	M	2019	South	Dumbéa		Wound	Lower limb	C. diphtheriae	Negative	NR	533	193 = SL533	322	ERS13588153	GCA_947533935
75	1002	FRC0752	F	2019	Islands	Lifou		Wound	Body trunk	C. diphtheriae	Negative	NR	416	82 = SL416	108	ERS13588154	GCA_947533705
76	1003	FRC0753	F	2019	Islands	Lifou		Wound	Lower limb	C. diphtheriae	Negative	NR	416	82 = SL416	108	ERS13588155	GCA_947533925
77	1004	FRC0754	M	2019	Islands	Lifou		Wound	Lower limb	C. diphtheriae	Negative	NR	228	43 = SL228	512	ERS13588156	GCA_947533825
78	1005	FRC0755	F	2019	Islands	Lifou		Urine	Urine	C. diphtheriae	Negative	NR	416	82 = SL416	108	ERS13588157	GCA_947533865
79	1006	FRC0756	M	2019	Islands	Lifou		Wound	Upper limb	C. diphtheriae	Negative	NR	416	82 = SL416	108	ERS13588158	GCA_947533835
80	1007	FRC0757	M	2019	South	Nouméa		Abscess	Upper limb	C. diphtheriae	Negative	NR	416	82 = SL416	108	ERS13588159	GCA_947533945
81	1075	FRC0758	M	2019	Islands	Ouvéa		Abscess	Lower limb	C. diphtheriae	Negative	NR	232	50 = SL232	122	ERS13588164	GCA_947533735
82	1008	FRC0759	M	2019	North	Kaala Gomen		Abscess	Upper limb	C. diphtheriae	Negative	NR	228	43 = SL228	511	ERS13588160	GCA_947533905
84	1009	FRC0760	M	2019	Islands	Maré		Wound	body trunk	C. diphtheriae	Negative	NR	228	43 = SL228	513	ERS13588161	GCA_947533895
85	1010	FRC0761	F	2019	South	Nouméa		Wound	Lower limb	C. diphtheriae	Negative	NR	416	82 = SL416	108	ERS13588162	GCA_947533685
86	1011	FRC0762	F	2019	NR	Vanuatu (not New Caledonia)	2	Throat	Throat	C. diphtheriae	Negative	NR	595	172 = SL595	514	ERS13588163	GCA_947533695

a NR: not relevant.

A large majority of cases (51/58, 87.9%) corresponded to polymicrobial wounds or abscesses, in association with S. aureus (35/58, 60.3%) and/or S. pyogenes (31/58, 53.4%) (Table S1). For the remaining patients, C. diphtheriae was found in 1 urine, 2 throat samples, 1 implantable site, and 3 blood cultures; the three latter isolates were found in pure culture. The urine sample was collected because of fever of unknown origin (but without any urinary functional sign). Positive blood cultures were associated with an infective endocarditis in 2 cases, while the third case with positive blood culture was probably a cutaneous contamination. All carriers of *tox*-positive C. diphtheriae were properly vaccinated.

### Microbiological characteristics.

The search of the *tox* gene by PCR showed that most isolates (52/58, 89.7%) were *tox*-negative. The six *tox*-positive isolates corresponded to cases imported from Vanuatu. Elek’s toxigenicity test showed that all of these isolates were toxigenic, i.e., produced the toxin (Table 1).

Antimicrobial susceptibility testing (Table S1) showed that all isolates were susceptible to amoxicillin, ciprofloxacin, rifampicin, co-trimoxazole, erythromycin, azithromycin, vancomycin and clindamycin (except one). For tetracycline, 17 resistant isolates were observed. Genomic analysis of antimicrobial resistance genes showed the presence of *tetO* gene in the 16 isolates of sublineage SL416 (Table S1). All isolates with *tetO* were resistant to tetracycline, and all isolates without *tetO* gene were susceptible to tetracycline except one (FRC0758); for this isolate, no explanation was found for its tetracycline resistance phenotype.

The interpretations for penicillin G depended on the reference system used: All isolates were resistant based on the 2019 CASFM reference system (Table S2), but using the 2013 CASFM guidelines, only 5 isolates were classified as intermediate, the others being classified as susceptible (Table S1).

The *pbp2m* gene was observed in one isolate (FRC0534), with decreased susceptibility to penicillin (CMI: 0.38 g/L). This gene is typically found on a small genetic unit, sometimes associated with *ermX*, in several plasmidic or chromosomal contexts, but is rare in C. diphtheriae populations (12, 20). Here, it was not associated with *ermX*, but with a nearby relaxase gene as described previously (12). No macrolide resistance gene was found among the studied isolates.

The genomic analyzes confirmed the MALDI-TOF species identification as C. diphtheriae for 57 isolates, whereas one *tox*-negative isolate (FRC0723) was identified as belonging to the closely related species *C. belfantii*. Biotyping showed that two isolates were of biovar Belfanti (including the *C. belfantii* isolate). Among the remaining isolates, 46 were of biovar Gravis and 10 of biovar Mitis (Table S1).

**FIG 2:**
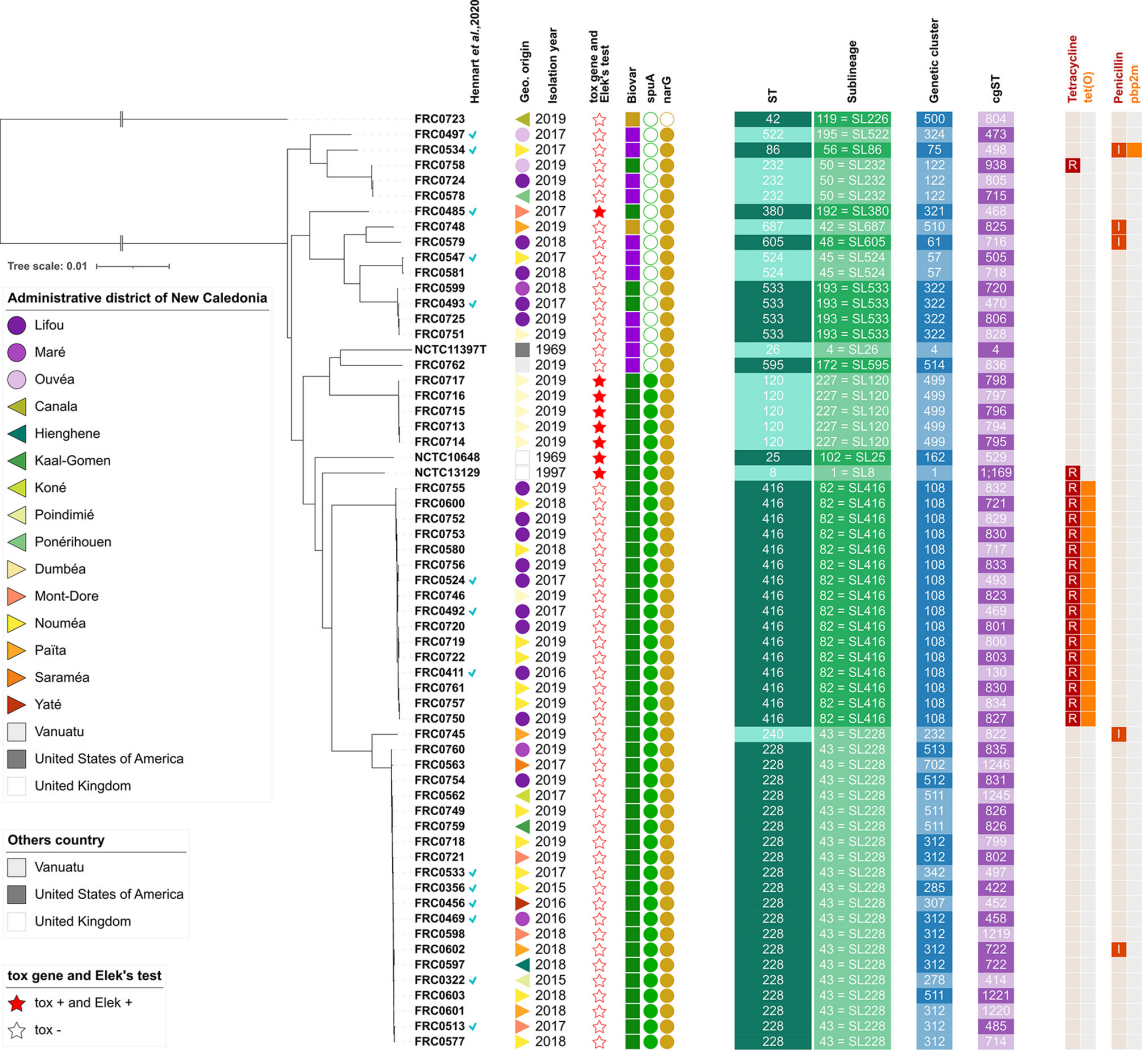
Diversity of Corynebacterium diphtheriae isolates from New Caledonia. The first column after the isolate identifier represents the locations of origin of the isolates (see color key). After the year of isolation column, the *tox*-negative isolates are represented by an empty star, and *tox*-positive ones by a full red star. The biovar is represented next: green for Gravis, purple for Mitis, brown for Belfanti. The following columns represent the presence of genes *spuA* (likely involved in glycogen metabolism; positive in biovar Gravis) and *narG* (involved in nitrate metabolism; positive in biovars Gravis and Mitis). On the four next columns, alternating dark and light colors indicate each sequence type (ST), sublineage (SL), genetic cluster (GC) or cgST change. Sublineages are given as: internal number = alias number (inherited, where possible, from 7-gene MLST numbers). The next column represents the tetracycline resistance phenotype: red for resistant (with R letter), gray for susceptible. The presence of the *tetO* gene is symbolized in orange on the next column, its absence in gray. The last two columns indicate in the same way, the penicillin phenotype and presence of gene pbp2m (I: intermediate).

Biovars Mitis and Gravis are distinguished by the ability to utilize glycogen. The *spuA* gene was reported as being associated with biovar Gravis isolates (12, 21). We confirmed a strong association between the presence of *spuA* and the Gravis phenotype: almost all (42/46; 91.3%) isolates having the *spuA* gene were of Gravis phenotype (Fig. 2). No molecular explanation was found for the ability to utilize glycogen of the four other Gravis isolates, and we noted that in some of them, the *spuA* gene was truncated (Table S1).

The nitrate reductase activity differentiates Mitis and Gravis isolates from biovar Belfanti isolates, which are nitrate-negative. As expected (22, 23), the *narG* gene (coding for nitrate reductase) was absent in the FRC0723 *C. belfantii* strain. However, no genomic explanation was found for the nitrate-negative phenotype of C. diphtheriae biovar Belfanti isolate (FRC0748).

### Genomic epidemiology of isolates.

C. diphtheriae isolates can be classified into 7-gene MLST sequence types (24) and into phylogenetic sublineages, which represent deep phylogenetic subdivisions of the population structure of this species that are highly concordant with ST classifications (25). The 7-gene MLST sequence type (ST) and cgMLST classifications into sublineages (SL; maximum of 500 mismatches out of 1305 loci) were defined for the 58 isolates (Table 1; Fig. 2). Based on MLST, there were 14 distinct STs, whereas cgMLST grouped the isolates into 13 different sublineages (SL). There was total agreement between ST and SL classifications, except that one isolate of sublineage SL228 had ST240 instead of ST228 (Table 1). Sublineage SL228 was represented by 21 isolates (36.2% of the total) and sublineage SL416 was represented by 16 (27.6%) isolates (Table 1). Hence, these two predominant sublineages represented more than half of the isolates circulating in New Caledonia. Isolates of these two sublineages were all of biovar Gravis. Whereas SL228 was isolated in 10 administrative districts, SL416 isolates were all recovered from Nouméa and Lifou except one (Table 1; Fig. 2).

Genomic clusters (GC) are much narrower genetic subdivisions of sublineages that have been defined as groups of isolates that have, among themselves, genetic distances (maximum of 25 cgMLST mismatches) compatible with outbreaks or recent transmission (25). Looking at this genotyping classification level, there were 22 distinct GCs (Table 1; Fig. 2). Two of these were recovered more than 8 times: GC108 (16 isolates) and GC312 (9 isolates), and they belonged to each of the predominant sublineages (SL416 and SL228), respectively. However, whereas SL228 comprised 9 other GCs, SL416 comprised only GC108, showing that SL416 is genetically very homogeneous, and may correspond to relatively recent transmission. In contrast, SL228 is genetically heterogeneous, in line with much wider geographic distribution across the island (Table 1). There was no documented epidemiological link between isolates within each of these two genomic clusters. Hence, they may correspond to cryptic transmission that was revealed here by genomic analyses.

Genetic cluster GC499 corresponded to *tox*-positive isolates from an index case and isolates from contacts that were screened during investigations around the index case (Investigation group 2 in Table 1). In this investigation, samples were collected in the same family in New Caledonia, of which some members had a recent history of travel to Vanuatu. Further comparisons of cgMLST allelic profiles within GC499 showed a maximum of 2 mismatches among the five isolates, strongly concordant with recent transmission of a single strain. The *tox* gene of these five strains had allele 21, with the deduced amino-acid replacement of a threonine by an isoleucine in the diphtheria toxin (compared to the reference from NCTC 13129, allele 1). Another contact isolate (case 86, isolate FRC0762) collected in Vanuatu during this investigation was in fact not related to the others based on genomic analyses: the isolate was *tox*-negative and belonged to a distinct sublineage (Table 1).

Another epidemiological investigation was triggered by a *tox*-negative isolate from a case on Lifou island. Upon screening of contacts, one isolate was found (see “Investigation group 1” in Table 1). Unexpectedly, the isolates from the index and the contact were in fact genetically unrelated, as they belong to different STs and sublineages (and of course, genetic clusters). The index isolate (FRC0492) belonged to the predominant genomic cluster circulating in Lifou island (GC108).

Genotypic data of the isolates from New Caledonia were compared with those of 876 global C. diphtheriae isolates available in public genome data repositories. Of the 13 sublineages from New Caledonia, 8 were also observed elsewhere (Table S3). Seven of these were observed in Australia, 2 in mainland France, and 3 in other European countries. Australian genomes represent only 66 (4.5%) of comparative genomes, and are therefore clearly overrepresented in sublineages shared with New Caledonia. Remarkably, two genomic clusters represented in New Caledonia, GC57 and GC61, were also reported 2012 to 2015 in Australia (Table S3), indicative of relatively recent transmission between the two islands.

## DISCUSSION

We provide clinical and microbiological data on a series of 58 infection or carriage cases of C. diphtheriae isolates in New Caledonia. Nearly 90% of the samples corresponded to skin infections, and C. diphtheriae was found in association with the pathogens S. aureus or S. pyogenes in over three quarters of these cases. Furthermore, the vast majority of C. diphtheriae isolates were nontoxigenic, which puts the pathogenicity of C. diphtheriae in skin infections into perspective. This study suggests that C. diphtheriae could be pathogenic itself or can colonize a preexisting infection caused by another bacterium. Coinfection could contribute to an increase of severity, compared to the same infection without C. diphtheriae. The classic clinical presentation is an ulcer covered with a pseudomembrane (26, 27). The pathogenicity of C. diphtheriae strains in this kind of context is not proved and the treatment should cover other pathogenic bacteria. The only two isolates from throat swabs were obtained from asymptomatic patients, in the scope of a confirmed case investigation. The strain of urinary origin was probably a contamination during the urine collection.

C. diphtheriae endocarditis is well described (28, 29). This infection frequently occurs in patients with predispositions, such as preexisting heart disease, prosthetic valves and intravenous drug use (29). Cases of C. diphtheriae endocarditis have been described in subjects who are generally younger than described for other pathogens. Endocarditis strains may or may not carry the diphtheria toxin gene, but cases of *tox*-negative endocarditis have predominated since high global vaccination coverage has been achieved (28, 29). Here, both cases of infective endocarditis were in young women with a history of rheumatic fever, a common condition in New Caledonia (30). People belonging to Melanesian and Polynesian populations, which are more affected by rheumatic fever than those of the European population, are at greater risk of developing infective endocarditis (30). Here, we showed that C. diphtheriae is a contributor; of note, six other cases of *tox*-negative C. diphtheriae endocarditis were previously reported in New Caledonia, five of them between 2005 and 2011, and one in 2021 (CHT laboratory data, unpublished), for a total of 8 cases, and with a history of acute rheumatoid fever for 6 patients.

A large majority of isolates were *tox*-negative strains. The only *tox*-positive isolates were from patients returning from Vanuatu, and were all toxigenic based on Elek’s test. Toxigenic isolates can cause severe respiratory or skin infections, and the action of the toxin can lead to cardiac dysfunction and neurological damage. In the present cases, no toxinic symptoms were observed.

Vaccination coverage among children is insufficient in Vanuatu, with only 62% of children being vaccinated in 2021 (31). Improving current vaccination coverage in Vanuatu would seem important, and a study of the epidemiological situation of diphtheria in this setting could guide control measures. Although carriers of *tox*-positive C. diphtheriae in New Caledonia were properly vaccinated, the immune response is directed against the toxin, and although it is effective against toxinic symptoms, it is not considered effective at preventing colonization.

The isolates were all susceptible to amoxicillin, and erythromycin, two first-line molecules for diphtheria treatment. For penicillin G, the French CA-SFM recommendations changed dramatically in 2014, in line with the European Committee on Antimicrobial Susceptibility Testing (EUCAST). These changes cover susceptibility testing protocols, disk loads used and interpretation thresholds. The new 1 IU disc load and 29 mm threshold classifies *de facto* all isolates as “resistant.” Conversely, the 2013 version classified almost all isolates in this study as “susceptible” and only a few as “intermediate.” Concerns about a similar change (breakpoint moved from 1 mg/L to 0.12 mg/L) in the Clinical and Laboratory Standards Institute (CLSI) interpretive thresholds have been expressed (32). There is a risk in overcategorizing isolates as penicillin-resistant, which would lead the clinician to use the broader spectrum macrolides. Moreover, the prescription of penicillin G appears appropriate to treat multimicrobial samples with Streptococcus pyogenes. According to the natural distribution of penicillin susceptibility values, the isolates from the present study should be considered to have a natural susceptibility phenotype (12, 32, 33).

Tetracycline resistance was observed in this study, concordant with other reports (12, 13, 34) but is clinically less relevant, given that recommended molecules such as amoxicillin were available. All isolates in which the *tetO* gene was found, were resistant to tetracycline. This gene encodes a protein that protects the ribosome from the action of tetracycline, allowing protein synthesis even in the presence of high intracellular tetracycline concentration (35).

The genotyping of isolates based on cgMLST provided confirmation or allowed to rule out suspected transmissions. In investigation group 1, the two isolates turned out to be phylogenetically very distant (as they belong to distinct sublineages), ruling-out transmission among the two cases. Instead of cross-transmission, the contact had asymptomatic carriage of a cocirculating *tox* negative strain. On the opposite, the isolates of investigation group 2 belonged to the same cgMLST genomic cluster (GC108). Although genomic clusters are defined using a tolerance of 25 mismatches, the isolates of this group were separated by only a maximum of 5 mismatches, underlying their very close genetic proximity.

Sublineage SL120, to which all five isolates in the *tox+* cluster (group 2) belong, was previously described only in two other isolates, one from 1995 in Finland and one isolated from 2015 in Australia; however, both belonged to other genomic clusters than GC499, which corresponds to group 2 isolates from New Caledonia. This illustrates the interest of classifying strains at both the sublineage and genomic group levels, as they provide information on genetic relationships, and hence transmission, at different timescales. The variant allele of the toxin gene found in the cluster of five *tox+* isolates (group 2) was previously described by Will et al. (36) as having a strong predicted structural impact on the toxin protein, and was found in only one isolate from Australia out of the 291 isolates analyzed by that previous study. We describe here 5 new isolates with this polymorphism.

Our genomic analyses uncovered a high genetic diversity of C. diphtheriae circulating in New Caledonia, with at least 13 different sublineages, consistent with the situation in other localities (11–14). This amount of diversity is remarkable considering the insular nature of New Caledonia. Nevertheless, nearly two-thirds of the isolates belonged to only two predominant sublineages, SL228 and SL416. Besides, the latter had very restricted genetic diversity, as it comprised a single genomic cluster. The high frequency of these genotypes could be related to the insular nature of the New Caledonia territory. The fact that all SL416 isolates belong to the same genomic cluster suggests relatively recent transmission at the population scale (perhaps a few decades). Interestingly, SL416 corresponded to the isolates carrying the *tetO* gene, conferring tetracycline resistance; whether this phenotype has favored the spread of this group is an interesting possibility, as observed for other bacteria (37). SL416 was previously reported only once, isolated in France from a patient returning from French Polynesia.

Sublineage SL228 represented 21 isolates (36.2% of our samples) and has already been described elsewhere, sporadically. Of all the published data available worldwide (876 genomes), nine strains outside New Caledonia belong to this sublineage (Table S3). Their provenances are as follows: An isolate from Romania in 1966, two Australian isolates, respectively, from 2013 and 2016, two mainland France isolates from 2011 and 2021, an isolate from Wallis and Futuna from 2012, and three isolates from French Polynesia, isolated in 2017, 2019 and 2020. As observed for other pathogens, e.g., Burkholderia pseudomallei (38), the predominant sublineages seem to be linked either to geographically close territories (Polynesia, Australia) or from metropolitan France, consistent with human transmission and dissemination.

In this study, cases with C. diphtheriae had an uneven geographic repartition across New Caledonia. This observation can in part be explained by the geographical distribution of the population: high population density in the main city of Nouméa (34.7% of the population) and in the South Province (74.9% of the population), but low density in the North Province and the Islands, with, respectively, 18.4% and 6.8% of the population (15). C. diphtheriae is more easily detected with MALDI-TOF mass spectrometry, with which only the laboratories in Nouméa are equipped, including the laboratory that processed the samples from Lifou island. Whereas the Lifou clinicians regularly took samples from wounds with unfavorable evolution in order to seek bacteriological documentation, the other centers did so much less systematically. This bias can contribute to the overrepresentation of cases in Lifou, despite a small population (9,195 inhabitants in 2019, i.e., 3.4% of the New Caledonian population) (15). This study therefore illustrates the difficulties of achieving a homogeneous diphtheria surveillance given existing heterogeneities of laboratory equipment, practices and awareness. In New Caledonia, the relatively high frequency of isolation of C. diphtheriae has led the local hospital reference laboratory to implement the PCR for the detection of the toxin gene on site, in order to accelerate the diagnostic of diphtheria, leading to apply more quickly and appropriately the recommendations of contact tracing, screening, and prophylaxis. Monitoring only symptomatic respiratory cases would have led to a very fragmentary picture, and the surveillance of diphtheria should clearly benefit from the addition of isolates from wound infections.

## MATERIALS AND METHODS

### Inclusion and exclusion criteria.

Local legislation obliges all laboratories of New Caledonia to report and send any isolated C. diphtheriae to the Centre Hospitalier Territorial (CHT) microbiology reference laboratory. Cases were defined as an infection or carrier with an isolate identified as C. diphtheriae, by the Matrix-Assisted Laser Desorption Ionization Time of Flight Mass Spectrometry (MALDI-TOF MS) automated system (Microflex Bruker). Isolation of all strains was done on the laboratories' usual agars, e.g., Columbia CNA (colistin-nalidixic acid) agar + 5% sheep blood (bioMérieux), as selective agars such as Tinsdale media are not being used. We investigated all episodes with C. diphtheriae isolated in culture (*n* = 57 from New Caledonia, plus one from Vanuatu; see below) over a 4 years period (from May 2015 to May 2019). The included cases were issued from clinical samples taken at the main hospital (CHT, *n* = 36) or from C. diphtheriae isolates sent to the reference Laboratory by other private and public laboratories located in New Caledonia (in this study, by two contributing laboratories; *n* = 21).

No clinical criteria for inclusion or exclusion were retained. In most cases (53/58), bacteriological examination was triggered by symptomatic manifestations. All initial cases (including the *tox*-negative ones, as investigations were triggered before the *tox* PCR result) were investigated after notification. The contact tracing was done by public health authorities by phone call to the patient and his/her physician, and if necessary an appointment was made to identify the high-risk contacts and to carry out the necessary sampling and vaccinations. Throat swabs were systematically taken for high-risk contacts, and skin lesions and wounds were systematically searched and swabbed if present. Prophylaxis was offered to all contacts of toxin-positive cases, and a vaccine booster for those not up to date, for all cases. In two investigations, further C. diphtheriae isolates were found: in investigation group 1 (Table 1), one *tox*-negative isolate (FRC0493) was found in a contact (case 29) of a *tox*-negative index case (case 28 in Table 1). In investigation group 2, four *tox*-positive isolates from contacts of an index case (case 60 in Table 1), detected by pharyngeal or skin lesion swabbing, and one *tox*-negative isolate (case 86, a contact in Vanuatu), were found. The latter case was included in the study, despite being from Vanuatu. Hence, 58 cases were included in total.

### Patient record and clinical data.

For each patient at CHT, a retrospective investigation in the medical records was carried out and relevant information was compiled in Table 1 (see also Table S1). For patients not recorded in the care software or whose diphtheria episode was not registered, a manual search in the records was carried out. For cases detected by laboratories outside the CHT, the prescribers were contacted in order to access the medical records. As the two dispensaries on the island of Lifou were the prescriber for a large number of cases, an on-site visit was organized and the missing data were recovered by consulting their records. Finally, the data collected by the Direction of Sanitary and Social Affairs (DASS) during each epidemiological investigation around a case, completed eventually the initial data.

### Antimicrobial susceptibility testing.

Antimicrobial susceptibility testing (AST) analyses were performed at the CHT laboratory after subculture of the strain on blood agar and verification of the identification by MALDI-TOF (Bruker). A 0.5 McFarland suspension was made in a saline solution (physiological serum), following the recommendations of the Comité de l’Antibiogramme de la Société Française de Microbiologie (CASFM), 2019 version. To determine the zone diameter for erythromycin and azithromycin, a 15 μg disk was used; for penicillin G, a 10 units disk content was used in addition to the disk with 1/10 dilution recommended by the CASFM 2013 version. The agar plates (Mueller-Hinton MHF, bioMérieux, Marcy l’Etoile, France) were inoculated by swabbing. After deposition of antibiotic paper discs (Bio-Rad) and Etest strips (bioMérieux, Marcy l’Etoile, France), the plates were incubated 18 to 24 h at 35 ± 2°C, under an atmosphere enriched with 5% CO_2_. The reading was performed manually, by the same operator, using a caliper. The names of antimicrobial agents, dosages, incubation conditions and interpretation thresholds are given in Table S2.

### Diphtheria toxin gene presence (*tox* positivity) and toxin production (toxigenicity).

All strains were tested for the presence of the *tox* gene at the CHT reference laboratory by real-time PCR, using the primers and probe from Schuhegger et al. (39): Cdiph-RTDT_Fw primer: 5′-TTA-TCA-AAA-GGT-TCG-GTG-ATG-GTG-3′; Cdiph-RTDT_rev2 primer: 5′-AAT-CTC-AAG-TTC-TAC-GCT-TAA-C-3′; and Cdiph-RTDT_So probe: 6-FAM-5’CGC-GTG-TAG-TGC-TCA-GCC-TTC-CCT-3′-TAMRA. Amplification conditions were modified by using the Roche LightCycler 2.0 (LC 2.0) thermal cycler, with a denaturation step (10 min, 95°C) and 45 cycles of amplification: denaturation for 15 s at 95°C and hybridization/elongation for 30 s at 60°C. For each assay, positive and negative controls were used. DNA extraction was carried out using MagNA Pure LC total Nucleic acid isolation kit on the automated MagNA Pure LC2.0 platform (Roche) from two to five colonies picked up from fresh cultured plates.

All isolates carrying the *tox* gene were tested at the national reference center at Institut Pasteur for toxigenicity, using the modified Elek immunoprecipitation test (40).

### Biovar determination.

Strains were characterized biochemically for pyrazinamidase, urease, and nitrate reductase and for utilization of maltose and trehalose using API Coryne strips (bioMérieux, Marcy l’Etoile, France) and the Rosco Diagnostica reagents (Eurobio, Les Ulis, France). The Hiss serum water test was used for glycogen fermentation. The biovar of isolates was determined based on the combination of nitrate reductase (positive in biovars Mitis and Gravis, negative in biovar Belfanti) and glycogen fermentation (positive in biovar Gravis only).

### Genome sequencing and phylogenetic analyses.

The DNA preparation, sequencing and the *de novo* assembly follows the same protocol as the previous studies (12, 25). The taxonomy of the isolates was confirmed by a MASH (v2.2) genomic distance lower than 0.05 with C. diphtheriae type strain NCTC11397^T^ or *C. belfantii* type strain FRC0043 ^T^.

We built a core genome multiple sequence alignment (cg-MSA) from the assembled genome sequences. For this, the genome sequences were annotated using PROKKA v1.14.5 (41) with default parameters, resulting in GFF files. Panaroo v1.2.3 (42) was used to define protein-coding gene clusters, with a threshold set at 80% amino acid identity, then the protein-coding gene sequences of each locus were aligned using MAFFT v7.467 (43) with default parameters. Core genes were defined as being present in 95% of genomes and were concatenated into a cg-MSA by Panaroo. IQtree version 2 (44) with best-fit model GTR+F+R7 was used to build a tree.

We used AMRFinderPlus v3.10.20 to identify known resistance genes in C. diphtheriae genomic sequences, based on the December 21^st^, 2021 database and using the following parameters to define presence of a gene: identity > 90% and length coverage > 50%. We used BLASTN (identity > 90% and coverage > 50%) to search for the presence of the *tox* gene, *spuA* gene (associated with biovar Gravis) and *narG* (associated with biovar Belfantii).

MLST and cgMLST genotypes were defined using the Institut Pasteur C. diphtheriae and C. ulcerans database at https://bigsdb.pasteur.fr/diphtheria. We used a previously described cgMLST scheme, database and associated classification schemes (25) for genomic clusters (25 mismatch threshold) and sublineages (500 mismatches). The isolates provenance data and genome sequences were imported into an isolates database (https://bigsdb.pasteur.fr/cgi-bin/bigsdb/bigsdb.pl?db=pubmlst_diphtheria_isolates&page=query). A publicly available BIGSdb project (i.e., a browsable list of isolates) entitled ‘New Caledonia 2015–2019 study’, corresponding to the present data set, was created to facilitate retrieval and analysis of the data. To enable the genotyping of the diphtheria toxin gene *tox*, the nomenclature of the toxin gene alleles from BIGSdb-Pasteur database was used: https://bigsdb.pasteur.fr/cgi-bin/bigsdb/bigsdb.pl?db=pubmlst_diphtheria_seqdef&page=schemeInfo&scheme_id=4.

### Ethical statement.

This work was approved by the CHT ethics committee (Avis 2022-001).

### Data statement.

The genomic sequences generated in this study were deposited in the ENA archive and are available from the INSDC databases (ENA/NCBI/DDBJ) under accession numbers ERS13588127 to ERS13588170 (see Tables 1 and S3).
