# Supplementary material for: Genomic Epidemiology of Corynebacterium diphtheriae in New Caledonia
Source: Microbiol Spectr. 2023 Apr 12;11(3):e04616-22. doi: 10.1128/spectrum.04616-22 (PMC10269643; doi:10.1128/spectrum.04616-22)
Supplement: Supplemental file 1 — Tables S1 to S3. Download spectrum.04616-22-s0001.pdf, PDF file, 1.4 MB [file spectrum.04616-22-s0001.pdf]

Supplementary appendix

Genomic epidemiology of *Corynebacterium diphtheriae* in New Caledonia

Eve Tessier[1, 2], Melanie Hennart[3,4], Edgar Badell [3,5], Virginie Passet [3,5], Julie Toubiana[3,5,6], Antoine Biron [2], Ann-Claire Gourinat [2], Audrey Merlet [7], Julien Colot[2,8] and Sylvain Brisse[3,5,*]

Affiliations

[1] CHU Nantes, Service de Bactériologie et des Contrôles Microbiologiques, Nantes, France.

[2] Microbiology Laboratory, Centre Hospitalier Territorial Gaston Bourret, Nouméa, New Caledonia

[3] Institut Pasteur, Université Paris Cité, Biodiversity and Epidemiology of Bacterial Pathogens, Paris, France.

[4] Sorbonne Université, Collège doctoral, F-75005 Paris, France

[5] National Reference Center for the Corynebacteria of the diphtheriae complex, Paris, France.

[6] Université Paris Cité, Department of General Pediatrics and Pediatric Infectious Diseases, Hôpital Necker–Enfants Malades, APHP, Paris, France

[7] Infectious diseases unit, Centre Hospitalier Territorial Gaston Bourret, Nouméa, New Caledonia

[8] Institut Pasteur de Nouvelle Calédonie, Groupe de Bactériologie médicale et environnementale Nouméa, New Caledonia

Table S1. Extended characteristics of the isolates.

Study ID	ID in BIGSdb	Isolate ID	cgST (cgMLST)	Biovar	Presence of *Staphylococcus aureus*	Presence of *Streptococcus pyogenes*	Other bacteria present:	Penicillin G 1	Penicillin G 10	Penicillin G E-Test	Ampicillin E-test	Ciprofloxacine	Clindamycin	Rifampicin	Cotrimoxazole	Tetracycline	Erythromycin	Azithromycin	Vancomycin	spuA	narG	tox in genome	TETRACYCLINE Resistance genes	BETA-LACTAM resistance genes
68	973	FRC0723	804	Belfanti	Yes	Yes	*P. mirabilis*	20 (R)	31 (S)	0.19 (R)	0.125 (S)	33 (S)	24 (S)	42 (S)	36 (S)	40 (S)	39 (S)	31 (S)	20 (S)	-	-	-	-	-
86	1011	FRC0762	836	Mitis	No	No		21 (R)	30 (S)	0.25 (R)	0.19 (S)	30 (S)	27 (S)	37 (S)	31 (S)	35 (S)	34 (S)	28 (S)	20 (S)	-	narG*	-	-	-
27	497	FRC0485	468	Gravis	No	Yes		21 (R)	34 (S)	0.19 (R)	0.25 (S)	33 (S)	23 (S)	38 (S)	35 (S)	40 (S)	40 (S)	33 (S)	20 (S)	-	narG*	tox_diphtheriae	-	-
29	500	FRC0493	470	Gravis	Yes	No		21 (R)	35 (S)	0.25 (R)	0.25 (S)	32 (S)	25 (S)	37 (S)	33 (S)	39 (S)	40 (S)	35 (S)	20 (S)	-	narG*	-	-	-
48	868	FRC0599	720	Gravis	Yes	No	*Streptococcus anginosus*	24 (R)	31 (S)	0.125 (S)	0.125 (S)	28 (S)	25 (S)	36 (S)	34 (S)	37 (S)	ND	31 (S)	19 (S)	-	narG*	-	-	-
70	975	FRC0725	806	Mitis	Yes	Yes		23 (R)	30 (S)	0.19 (R)	0.19 (S)	32 (S)	23 (S)	38 (S)	32 (S)	35 (S)	36 (S)	30 (S)	20 (S)	-	narG*	-	-	-
73	1001	FRC0751	828	Mitis	Yes	Yes	*Arcanobacterium haemolyticum*	27 (R)	32 (S)	0.125 (S)	0.125 (S)	33 (S)	24 (S)	38 (S)	34 (S)	38 (S)	36 (S)	28 (S)	20 (S)	-	narG*	-	-	-
30	503	FRC0497	473	Mitis	No	Yes	*Providencia rettgeri* and *Pseudo*	21 (R)	30 (S)	0.19 (R)	0.25 (S)	30 (S)	22 (S)	33 (S)	32 (S)	33 (S)	38 (S)	30 (S)	19 (S)	-	narG*	-	-	-

							monas aeruginosa																	
60	962	FRC0713	794	Gravis	Yes	Yes		21 (R)	32 (S)	0.19 (R)	0.19 (S)	30 (S)	22 (S)	35 (S)	33 (S)	33 (S)	31 (S)	25 (S)	19 (S)	spuA*	narG*	tox_diphtheriae*	-	-
61	963	FRC0714	795	Gravis	Yes	Yes		19 (R)	31 (S)	0.19 (R)	0.19 (S)	30 (S)	22 (S)	34 (S)	33 (S)	35 (S)	31 (S)	28 (S)	18 (S)	spuA*	narG*	tox_diphtheriae*	-	-
63	964	FRC0715	796	Gravis	Yes	No		20 (R)	32 (S)	0.19 (R)	0.19 (S)	31 (S)	22 (S)	35 (S)	32 (S)	33 (S)	33 (S)	29 (S)	18 (S)	spuA*	narG*	tox_diphtheriae*	-	-
64	965	FRC0716	797	Gravis	No	No		21 (R)	32 (S)	0.19 (R)	0.19 (S)	32 (S)	22 (S)	35 (S)	33 (S)	33 (S)	35 (S)	29 (S)	19 (S)	spuA*	narG*	tox_diphtheriae*	-	-
66	967	FRC0717	798	Gravis	Yes	Yes		19 (R)	32 (S)	0.19 (R)	0.19 (S)	30 (S)	22 (S)	34 (S)	32 (S)	32 (S)	33 (S)	27 (S)	17 (S)	spuA*	narG*	tox_diphtheriae*	-	-
57	998	FRC0748	825	Belfanti	Yes	No		20 (R)	28 (I)	0.25 (R)	0.38 (S)	29 (S)	24 (S)	35 (S)	26 (S)	37 (S)	33 (S)	28 (S)	19 (S)	-	narG*	-	-	-
53	994	FRC0745	822	Gravis	Yes	No	Streptococcus dysgalactiae	22 (R)	27 (I)	0.125 (S)	0.19 (S)	25 (S)	24 (S)	34 (S)	33 (S)	34 (S)	34 (S)	29 (S)	18 (S)	spuA*	narG*	-	-	-
17	434	FRC0322	414	Gravis	No	Yes	*E. coli*	20 (R)	32 (S)	0.19 (R)	0.125 (S)	29 (S)	23 (S)	36 (S)	33 (S)	33 (S)	34 (S)	28 (S)	19 (S)	spuA?-34%	narG*	-	-	-
20	442	FRC0356	422	Gravis	No	No	*Streptococcus dygalactiae* and *Acinetobacter baumannii*	21 (R)	30 (S)	0.19 (R)	0.25 (S)	30 (S)	20 (S)	34 (S)	32 (S)	32 (S)	34 (S)	28 (S)	18 (S)	spuA?-34%	narG*	-	-	-

24	478	FRC0456	452	Gravis	No	No	*Vibrio alginolyticus* and *S. dysgalactiae*	23 (R)	36 (S)	0.094 (S)	0.125 (S)	30 (S)	26 (S)	34 (S)	34 (S)	34 (S)	36 (S)	33 (S)	19 (S)	spuA?-34%	narG*	-	-	-
25	484	FRC0469	458	Gravis	Yes	No	*S. dysgalactiae B*	23 (R)	36 (S)	0.125 (S)	0.125 (S)	30 (S)	27 (S)	35 (S)	33 (S)	37 (S)	40 (S)	35 (S)	20 (S)	spuA?-44%	narG*	-	-	-
31	515	FRC0513	485	Gravis	No	No		15 (R)	29 (S)	0.38 (R)	0.5 (S)	29 (S)	24 (S)	35 (S)	32 (S)	35 (S)	35 (S)	31 (S)	19 (S)	spuA?-44%	narG*	-	-	-
41	861	FRC0577	714	Gravis	Yes	No		23 (R)	36 (S)	0.19 (R)	0.19 (S)	32 (S)	26 (S)	39 (S)	33 (S)	39 (S)	40 (S)	35 (S)	21 (S)	spuA?-44%	narG*	-	-	-
46	1426	FRC0597	722	Gravis	No	No	*Streptococcus agalactiae* and *Proteus vulgaris*	23 (R)	30 (S)	0.19 (R)	0.38 (S)	26 (S)	22 (S)	35 (S)	34 (S)	34 (S)	35 (S)	32 (S)	19 (S)	spuA?-44%	narG*	-	-	-
47	1427	FRC0598	1219	Gravis	No	No	*Streptococcus* agalactiae, *E.coli, P. aeruginosa*	24 (R)	30 (S)	0.125 (S)	0.19 (S)	28 (S)	24 (S)	36 (S)	35 (S)	36 (S)	38 (S)	30 (S)	19 (S)	spuA?-44%	narG*	-	-	-
50	1428	FRC0601	1220	Gravis	Yes	Yes		23 (R)	37 (S)	0.25 (R)	0.125 (S)	33 (S)	28 (S)	39 (S)	36 (S)	36 (S)	39 (S)	33 (S)	21 (S)	spuA?-44%	narG*	-	-	-
51	870	FRC0602	722	Gravis	Yes	Yes		22 (R)	27 (I)	0.19 (R)	0.25 (S)	28 (S)	23 (S)	36 (S)	34 (S)	34 (S)	30 (S)	28 (S)	19 (S)	spuA?-44%	narG*	-	-	-

65	971	FRC0721	802	Gravis	No	Yes		20 (R)	32 (S)	0.19 (R)	0.19 (S)	30 (S)	23 (S)	35 (S)	31 (S)	31 (S)	33 (S)	29 (S)	20 (S)	spuA?-44%	narG*	-	-	-
74	968	FRC0718	799	Gravis	Yes	Yes		23 (R)	30 (S)	0.19 (R)	0.25 (S)	29 (S)	24 (S)	35 (S)	29 (S)	32 (S)	38 (S)	ND	19 (S)	spuA?-44%	narG*	-	-	-
36	527	FRC0533	497	Gravis	Yes	Yes		23 (R)	32 (S)	0.19 (R)	0.25 (S)	28 (S)	24 (S)	34 (S)	34 (S)	34 (S)	36 (S)	29 (S)	19 (S)	spuA?-34%	narG*	-	-	-
40	1453	FRC0562	1245	Gravis	No	No		22 (R)	30 (S)	0.125 (S)	0.19 (S)	27 (S)	23 (S)	34 (S)	34 (S)	34 (S)	35 (S)	30 (S)	19 (S)	spuA?-34%	narG*	-	-	-
52	1429	FRC0603	1221	Gravis	No	No		26 (R)	31 (S)	0.094 (S)	0.094 (S)	32 (S)	24 (S)	38 (S)	32 (S)	38 (S)	37 (S)	29 (S)	18 (S)	spuA?-34%	narG*	-	-	-
58	999	FRC0749	826	Gravis	No	Yes		22 (R)	30 (S)	0.19 (R)	0.19 (S)	30 (S)	23 (S)	36 (S)	32 (S)	36 (S)	35 (S)	26 (S)	19 (S)	spuA?-34%	narG*	-	-	-
82	1008	FRC0759	826	Gravis	No	Yes	*Streptococcus agalactiae*	21 (R)	31 (S)	0.19 (R)	0.125 (S)	33 (S)	21 (S)	38 (S)	34 (S)	37 (S)	37 (S)	31 (S)	19 (S)	spuA?-34%	narG*	-	-	-
77	1004	FRC0754	831	Gravis	Yes	Yes		23 (R)	32 (S)	0.125 (S)	0.125 (S)	32 (S)	24 (S)	37 (S)	35 (S)	37 (S)	36 (S)	28 (S)	20 (S)	spuA?-34%	narG*	-	-	-
84	1009	FRC0760	835	Gravis	Yes	No		23 (R)	31 (S)	0.19 (R)	0.125 (S)	35 (S)	24 (S)	38 (S)	35 (S)	37 (S)	36 (S)	30 (S)	20 (S)	spuA?-34%	narG*	-	-	-

39	1454	FRC0563	1246	Gravis	Yes	No		24 (R)	30 (S)	0.19 (R)	0.19 (S)	33 (S)	22 (S)	33 (S)	32 (S)	33 (S)	33 (S)	29 (S)	19 (S)	spuA?-34%	narG*	-	-	-
38	536	FRC0547	505	Mitis	Yes	Yes	polymorphic flora	23 (R)	34 (S)	0.19 (R)	0.25 (S)	30 (S)	28 (S)	39 (S)	25 (S)	41 (S)	39 (S)	35 (S)	21 (S)	-	narG*	-	-	-
45	865	FRC0581	718	Mitis	Yes	Yes		21 (R)	29 (S)	0.19 (R)	0.25 (S)	27 (S)	24 (S)	35 (S)	19 (S)	37 (S)	31 (S)	28 (S)	19 (S)	-	narG*	-	-	-
44	863	FRC0579	716	Mitis	ND	ND		20 (R)	27 (I)	0.25 (R)	0.38 (S)	29 (S)	22 (S)	35 (S)	32 (S)	35 (S)	34 (S)	28 (S)	18 (S)	-	narG*	-	-	-
43	862	FRC0578	715	Mitis	No	Yes		23 (R)	29 (S)	0.125 (S)	0.19 (S)	29 (S)	23 (S)	34 (S)	32 (S)	38 (S)	32 (S)	28 (S)	19 (S)	-	narG*	-	-	-
69	974	FRC0724	805	Mitis	Yes	No	*S. dysgalactiae spp dysgalactiae*	20 (R)	30 (S)	0.125 (S)	0.125 (S)	28 (S)	24 (S)	36 (S)	32 (S)	36 (S)	36 (S)	28 (S)	20 (S)	-	narG*	-	-	-
81	1075	FRC0758	938	Gravis	Yes	No		20 (R)	30 (S)	0.38 (R)	0.25 (S)	31 (S)	24 (S)	36 (S)	31 (S)	18 (R)	32 (S)	27 (S)	20 (S)	-	narG*	-	-	-
37	528	FRC0534	498	Mitis	Yes	No	*Proteus mirabilis, Staphylococcus simulans, Staphylococcus pettenkoferi, Staphylococcus aureus,* and	16 (R)	27 (I)	0.38 (R)	0.125 (S)	29 (S)	24 (S)	33 (S)	32 (S)	32 (S)	36 (S)	29 (S)	19 (S)	-	narG*	-	-	pbp2m

							Corynebacterium stiratum.																	
22	140	FRC0411	130	Gravis	No	Yes		19 (R)	30 (S)	0.25 (R)	0.25 (S)	29 (S)	19 (R)	34 (S)	24 (S)	15 (R)	33 (S)	27 (S)	18 (S)	spuA*	narG*	-	tet(O)	-
28	499	FRC0492	469	Gravis	Yes	Yes		20 (R)	34 (S)	0.125 (S)	0.25 (S)	31 (S)	24 (S)	37 (S)	31 (S)	22 (R)	38 (S)	33 (S)	20 (S)	spuA*	narG*	-	tet(O)	-
35	523	FRC0524	493	Gravis	Yes	No		21 (R)	30 (S)	0.25 (R)	0,38 (S)	30 (S)	24 (S)	33 (S)	30 (S)	22 (R)	34 (S)	30 (S)	19 (S)	spuA*	narG*	-	tet(O)	-
42	864	FRC0580	717	Gravis	Yes	Yes		22 (R)	33 (S)	0.19 (R)	0.19 (S)	30 (S)	25 (S)	37 (S)	33 (S)	23 (R)	38 (S)	31 (S)	20 (S)	spuA*	narG*	-	tet(O)	-
49	869	FRC0600	721	Gravis	No	No		21 (R)	29 (S)	0.25 (R)	0.25 (S)	28 (S)	21 (S)	35 (S)	27 (S)	17 (R)	30 (S)	27 (S)	18 (S)	spuA*	narG*	-	tet(O)	-
54	996	FRC0746	823	Gravis	Yes	Yes		21 (R)	34 (S)	0.19 (R)	0.19 (S)	32 (S)	24 (S)	37 (S)	33 (S)	22 (R)	39 (S)	31 (S)	20 (S)	spuA*	narG*	-	tet(O)	-
59	969	FRC0719	800	Gravis	No	No		22 (R)	31 (S)	0.19 (R)	0.19 (S)	34 (S)	23 (S)	37 (S)	30 (S)	19 (R)	34 (S)	29 (S)	19 (S)	spuA*	narG*	-	tet(O)	-
62	970	FRC0720	801	Gravis	Yes	Yes	Streptococcus dysgalactiae	20 (R)	30 (S)	0.25 (R)	0.19 (S)	31 (S)	23 (S)	36 (S)	30 (S)	18 (R)	33 (S)	29 (S)	20 (S)	spuA*	narG*	-	tet(O)	-
67	972	FRC0722	803	Gravis	No	Yes		20 (R)	29 (S)	0.25 (R)	0.19 (S)	26 (S)	22 (S)	30 (S)	24 (S)	17 (R)	31 (S)	25 (S)	18 (S)	spuA*	narG*	-	tet(O)	-
72	1000	FRC0750	827	Gravis	Yes	Yes		21 (R)	30 (S)	0.25 (R)	0.25 (S)	28 (S)	23 (S)	36 (S)	30 (S)	19 (R)	35 (S)	30 (S)	20 (S)	spuA*	narG*	-	tet(O)	-

75	1002	FRC0752	829	Gravis	Yes	No	22 (R)	30 (S)	0.25 (R)	0.19 (S)	30 (S)	24 (S)	35 (S)	30 (S)	18 (R)	34 (S)	27 (S)	19 (S)	spuA*	narG*	-	tet(O)	-
76	1003	FRC0753	830	Gravis	ND	ND	22 (R)	31 (S)	0.25 (R)	0.25 (S)	25 (S)	23 (S)	37 (S)	29 (S)	20 (R)	35 (S)	29 (S)	19 (S)	spuA*	narG*	-	tet(O)	-
78	1005	FRC0755	832	Gravis	No	No	21 (R)	31 (S)	0.25 (R)	0.25 (S)	31 (S)	23 (S)	36 (S)	30 (S)	20 (R)	36 (S)	27 (S)	19 (S)	spuA*	narG*	-	tet(O)	-
79	1006	FRC0756	833	Gravis	Yes	Yes	21 (R)	29 (S)	0.38 (R)	0.25 (S)	28 (S)	24 (S)	36 (S)	32 (S)	18 (R)	35 (S)	28 (S)	20 (S)	spuA*	narG*	-	tet(O)	-
80	1007	FRC0757	834	Gravis	No	No	22 (R)	29 (S)	0.38 (R)	0.25 (S)	27 (S)	22 (S)	36 (S)	30 (S)	18 (R)	35 (S)	29 (S)	19 (S)	spuA*	narG*	-	tet(O)	-
85	1010	FRC0761	830	Gravis	Yes	Yes	19 (R)	29 (S)	0.38 (R)	0.25 (S)	25 (S)	24 (S)	36 (S)	31 (S)	18 (R)	32 (S)	29 (S)	19 (S)	spuA*	narG*	-	tet(O)	-

NR: not relevant; ND: not documented

*: more than 90% identity and 100% coverage with the reference sequence

?-xx%: xx% of the protein is truncated.

Table S2. Antibiotics susceptibility testing conditions.

Conditions			Diameter		MIC (E-test)	
**Suspension**	**Disk dosage**	**Antimicrobial agent**	**S ≥**	**R<**	**S**	**R**
**0.5 McF**	E-test	Ampicillin			≤2	>8
	E-test	Penicillin G			≤0.125	>0.125
	1 UI	Penicillin G	29	29		
	5 µg	Vancomycin	17	17		
	5 µg	Ciprofloxacin	25	25		
	2 µg	Clindamycin	20	20		
	1.25 µg - 23.75 µg	Cotrimoxazole	19	16		
	30 µg	Tetracycline	24	24		
	5 µg	Rifampicin	30	25		
**0.5 McF/10th dilution**	15 µg	Erythromycin	22	17		
	15 µg	Azithromycin	22	17		
	10 UI	Penicillin G	29	18		

Table S3. Origins and characteristics of *C. diphtheriae* isolates that belong to sublineages sampled in New Caledonia

id	isolate	Other isolate name	cgST (cgMLST)	ST (MLST)	Genomic_cluster	Sublineage alias #	Sublineage (internal to BIGSdb)	species	biovar	tox_gene_PCR	tox_production_Elek	isolation_year	city	country	continent	state_or_locality	travel_history	source	infection_or_disease	accession_number
423	FRC0290		403	86	75	SL10007	56	C. diphtheriae	Mitis	Negative		2015	Noumea	New Caledonia	Oceania	New Caledonia	Not documented	Cutaneous	Not documented	ERS4330901
528	FRC0534		498	86	75	SL10007	56	C. diphtheriae	Mitis	Negative		2017	Noumea	New Caledonia	Oceania	New Caledonia	Not documented	Cutaneous	Abscess	ERS4330982
933	SRR6816571	CD4	777	86	75	SL10007	56	C. diphtheriae	Mitis			2008		Australia	Oceania		Not documented	Cutaneous (leg)		PRJNA436425
590	CIP107570		556	127	387	SL120#	227	C. diphtheriae	Gravis	Positive	Positive	1995	Tamper	Finland	Europe		Not documented			ERS4330828
928	SRR6816605	CD29	772	120	486	SL120#	227	C. diphtheriae	Gravis			2015		Australia	Oceania		Not documented	Cutaneous (foot)		PRJNA436425
962	FRC0713		794	120	499	SL120#	227	C. diphtheriae	Gravis	Positive	Positive	2019	Noumea	New Caledonia	Oceania	New Caledonia	Travel from Vanuatu	Cutaneous	Wound	ERS13588135
963	FRC0714		795	120	499	SL120#	227	C. diphtheriae	Gravis	Positive	Positive	2019	Noumea	New Caledonia	Oceania	New Caledonia	Travel from Vanuatu	Cutaneous	Wound	ERS13588136
964	FRC0715		796	120	499	SL120#	227	C. diphtheriae	Gravis	Positive	Positive	2019	Noumea	New Caledonia	Oceania	New Caledonia	Travel from Vanuatu	Cutaneous	Wound	ERS13588137
965	FRC0716		797	120	499	SL120#	227	C. diphtheriae	Gravis	Positive	Positive	2019	Noumea	New Caledonia	Oceania	New Caledonia	Not documented	Throat	Throat	ERS13588138

967	FRC0717		798	120	499	SL120#	227	C. diphtheriae	Gravis	Positive	Positive	2019	Noumea	New Caledonia	Oceania	New Caledonia	Not documented	Cutaneous	Wound	ERS13588139
83	SRR6816576	CD9	77	745	64	SL18	50	C. diphtheriae	Mitis			2012		Australia	Oceania		Not documented	Cutaneous (unknown site)		PRJNA436425
751	KL0816		629	499	413	SL18	50	C. diphtheriae	Mitis			2016	Bremerhaven	Germany	Europe		Not documented	leg	wound infection	PRJNA416260
862	FRC0578		715	232	122	SL18	50	C. diphtheriae	Mitis	Negative		2018	Noumea	New Caledonia	Oceania	New Caledonia	None	Cutaneous	Abscess	ERS13588128
974	FRC0724		805	232	122	SL18	50	C. diphtheriae	Mitis	Negative		2019	Noumea	New Caledonia	Oceania	New Caledonia	Not documented	Cutaneous	Wound	ERS13588146
1075	FRC0758		938	232	122	SL18	50	C. diphtheriae	Gravis	Negative		2019	Noumea	New Caledonia	Oceania	New Caledonia	Not documented	Cutaneous	Abscess	ERS13588164
973	FRC0723		804	42	500	SL226#	119	**C. belfantii**	Belfanti	Negative		2019	Noumea	New Caledonia	Oceania	New Caledonia	Not documented	Cutaneous	Wound	ERS13588145
73	SRR6816563	CD44	67	240	54	SL228	43	C. diphtheriae	Gravis			2016		Australia	Oceania		Not documented	Cutaneous (wound unknown site)		PRJNA436425
84	SRR6816577	CD100	78	240	65	SL228	43	C. diphtheriae	Gravis			2013		Australia	Oceania		Not documented	Cutaneous (leg)		PRJNA436425
369	FRC0084		350	228	224	SL228	43	C. diphtheriae	Gravis	Negative	Negative	2011	Saint Gaudens	France	Europe	Mainland France	Not documented	Respiratory	Not documented	ERS4330857

377	FRC0104	358	240	232	SL228	43	C. diphtheriae	Gravis	Negative		2012	Mata Utu	French Polynesia	Oceania	Wallis and Futuna	Not documented	Cutaneous	Not documented	ERS4330865
434	FRC0322	414	228	278	SL228	43	C. diphtheriae	Gravis	Negative		2015	Noumea	New Caledonia	Oceania	New Caledonia	Not documented	Cutaneous	Abscess	ERS4330911
438	FRC0332	418	228	282	SL228	43	C. diphtheriae	Gravis	Negative		2015	Noumea	New Caledonia	Oceania	New Caledonia	Not documented	Cutaneous	Not documented	ERS4330914
442	FRC0356	422	228	285	SL228	43	C. diphtheriae	Gravis	Negative		2015	Noumea	New Caledonia	Oceania	New Caledonia	Not documented	Cutaneous	Wound	ERS4330918
474	FRC0449	449	228	282	SL228	43	C. diphtheriae	Gravis	Negative		2016	Noumea	New Caledonia	Oceania	New Caledonia	Not documented	Cutaneous	Not documented	ERS4330948
478	FRC0456	452	228	307	SL228	43	C. diphtheriae	Gravis	Negative		2016	Noumea	New Caledonia	Oceania	New Caledonia	Not documented	Cutaneous	Wound	ERS4330949
484	FRC0469	458	228	312	SL228	43	C. diphtheriae	Gravis	Negative		2016	Noumea	New Caledonia	Oceania	New Caledonia	Not documented	Cutaneous	Wound	ERS4330954
515	FRC0513	485	228	312	SL228	43	C. diphtheriae	Gravis	Negative		2017	Noumea	New Caledonia	Oceania	New Caledonia	Not documented	Cutaneous	Abscess	ERS4330974
516	FRC0514	486	228	312	SL228	43	C. diphtheriae	Gravis	Negative		2017	Noumea	New Caledonia	Oceania	New Caledonia	Not documented	Cutaneous	Not documented	ERS4330975
527	FRC0533	497	228	342	SL228	43	C. diphtheriae	Gravis	Negative		2017	Noumea	New Caledonia	Oceania	New Caledonia	Not documented	Cutaneous	Wound	ERS4330981
585	CIP107565	552	145	383	SL228	43	C. diphtheriae	Gravis	Negative		1966	Suceava	Romania	Europe		Not documented			ERS4330824

861	FRC0577		714	228	312	SL228	43	C. diphtheriae	Gravis	Negative		2017	Noumea	New Caledonia	Oceania	New Caledonia	None	Cutaneous	Wound	ERS13588127
870	FRC0602		722	228	312	SL228	43	C. diphtheriae	Gravis	Negative		2018	Noumea	New Caledonia	Oceania	New Caledonia	None	Cutaneous	Wound	ERS13588134
968	FRC0718		799	228	312	SL228	43	C. diphtheriae	Gravis	Negative		2019	Noumea	New Caledonia	Oceania	New Caledonia	Not documented	Cutaneous	Abscess	ERS13588140
971	FRC0721		802	228	312	SL228	43	C. diphtheriae	Gravis	Negative		2019	Noumea	New Caledonia	Oceania	New Caledonia	Not documented	Cutaneous	Abscess	ERS13588143
994	FRC0745		822	240	232	SL228	43	C. diphtheriae	Gravis	Negative		2019	Noumea	New Caledonia	Oceania	New Caledonia	Not documented	Cutaneous	Abscess	ERS13588148
999	FRC0749		826	228	511	SL228	43	C. diphtheriae	Gravis	Negative		2019	Noumea	New Caledonia	Oceania	New Caledonia	Not documented	Cutaneous	Wound	ERS13588151
1004	FRC0754		831	228	512	SL228	43	C. diphtheriae	Gravis	Negative		2019	Noumea	New Caledonia	Oceania	New Caledonia	Not documented	Cutaneous	Abscess	ERS13588156
1008	FRC0759		826	228	511	SL228	43	C. diphtheriae	Gravis	Negative		2019	Noumea	New Caledonia	Oceania	New Caledonia	Not documented	Cutaneous	Abscess	ERS13588160
1009	FRC0760		835	228	513	SL228	43	C. diphtheriae	Gravis	Negative		2019	Noumea	New Caledonia	Oceania	New Caledonia	Not documented	Cutaneous	Wound	ERS13588161
1426	FRC0597		722	228	312	SL228	43	C. diphtheriae	Gravis	Negative		2018	Noumea	New Caledonia	Oceania	New Caledonia	Not documented	Cutaneous	Abscess	ERS13588165
1427	FRC0598		1219	228	312	SL228	43	C. diphtheriae	Gravis	Negative		2018	Noumea	New Caledonia	Oceania	New Caledonia	Not documented	Cutaneous	Abscess	ERS13588166

1428	FRC0601	1220	228	312	SL228	43	C. diphtheriae	Gravis	Negative		2018	Noumea	New Caledonia	Oceania	New Caledonia	Not documented	Cutaneous	Wound	ERS13588167
1429	FRC0603	1221	228	511	SL228	43	C. diphtheriae	Gravis	Negative		2018	Noumea	New Caledonia	Oceania	New Caledonia	Not documented	Cutaneous	Wound	ERS13588168
1453	FRC0562	1245	228	511	SL228	43	C. diphtheriae	Gravis	Negative		2017	Noumea	New Caledonia	Oceania	New Caledonia	Not documented	Blood	Infectious endocarditis on pacemaker	ERS13588169
1454	FRC0563	1246	228	702	SL228	43	C. diphtheriae	Gravis	Negative		2017	Noumea	New Caledonia	Oceania	New Caledonia	Not documented	Cutaneous	Abscess	ERS13588170
497	FRC0485	468	380	321	SL380#	192	C. diphtheriae	Gravis	Positive	Positive	2017	Noumea	New Caledonia	Oceania	New Caledonia	Travel from Vanuatu	Cutaneous	Wound	ERS4330965
140	FRC0411	130	416	108	SL416#	82	C. diphtheriae	Gravis	Negative		2016	Noumea	New Caledonia	Oceania	New Caledonia	None	Cutaneous	Wound	ERS4330931
499	FRC0492	469	416	108	SL416#	82	C. diphtheriae	Gravis	Negative		2017	Noumea	New Caledonia	Oceania	New Caledonia	Not documented	Cutaneous	Wound	ERS4330967
523	FRC0524	493	416	108	SL416#	82	C. diphtheriae	Gravis	Negative		2017	Noumea	New Caledonia	Oceania	New Caledonia	Not documented	Cutaneous	Wound	ERS4330979
864	FRC0580	717	416	108	SL416#	82	C. diphtheriae	Gravis	Negative		2018	Noumea	New Caledonia	Oceania	New Caledonia	None	Cutaneous	Wound	ERS13588130
869	FRC0600	721	416	108	SL416#	82	C. diphtheriae	Gravis	Negative		2018	Noumea	New Caledonia	Oceania	New Caledonia	None	Blood	Blood	ERS13588133
969	FRC0719	800	416	108	SL416#	82	C. diphtheriae	Gravis	Negative		2019	Noumea	New Caledonia	Oceania	New Caledonia	Not documented	Blood	Blood	ERS13588141

970	FRC0720		801	416	108	SL416#	82	C. diphtheriae	Gravis	Negative		2019	Noumea	New Caledonia	Oceania	New Caledonia	Not documented	Cutaneous	Wound	ERS13588142
972	FRC0722		803	416	108	SL416#	82	C. diphtheriae	Gravis	Negative		2019	Noumea	New Caledonia	Oceania	New Caledonia	Not documented	Cutaneous	Abscess	ERS13588144
996	FRC0746		823	416	108	SL416#	82	C. diphtheriae	Gravis	Negative		2019	Noumea	New Caledonia	Oceania	New Caledonia	Not documented	Cutaneous	Chronic wound foot	ERS13588149
1000	FRC0750		827	416	108	SL416#	82	C. diphtheriae	Gravis	Negative		2019	Noumea	New Caledonia	Oceania	New Caledonia	Not documented	Not documented	Abscess	ERS13588152
1002	FRC0752		829	416	108	SL416#	82	C. diphtheriae	Gravis	Negative		2019	Noumea	New Caledonia	Oceania	New Caledonia	Not documented	Cutaneous	Wound	ERS13588154
1003	FRC0753		830	416	108	SL416#	82	C. diphtheriae	Gravis	Negative		2019	Noumea	New Caledonia	Oceania	New Caledonia	Not documented	Cutaneous	Wound	ERS13588155
1005	FRC0755		832	416	108	SL416#	82	C. diphtheriae	Gravis	Negative		2019	Noumea	New Caledonia	Oceania	New Caledonia	Not documented	Urines	Urines	ERS13588157
1006	FRC0756		833	416	108	SL416#	82	C. diphtheriae	Gravis	Negative		2019	Noumea	New Caledonia	Oceania	New Caledonia	Not documented	Cutaneous	Wound	ERS13588158
1007	FRC0757		834	416	108	SL416#	82	C. diphtheriae	Gravis	Negative		2019	Noumea	New Caledonia	Oceania	New Caledonia	Not documented	Cutaneous	Abscess	ERS13588159
1010	FRC0761		830	416	108	SL416#	82	C. diphtheriae	Gravis	Negative		2019	Noumea	New Caledonia	Oceania	New Caledonia	Not documented	Cutaneous	Wound	ERS13588162
503	FRC0497		473	522	324	SL522#	195	C. diphtheriae	Mitis	Negative		2017	Noumea	New Caledonia	Oceania	New Caledonia	Not documented	Cutaneous	Wound	ERS4330969

500	FRC0493		470	533	322	SL533#	193	C. diphtheriae	Gravis	Negative		2017	Noumea	New Caledonia	Oceania	New Caledonia	Not documented	Cutaneous	Wound	ERS4330968
868	FRC0599		720	533	322	SL533#	193	C. diphtheriae	Gravis	Negative		2018	Noumea	New Caledonia	Oceania	New Caledonia	None	Cutaneous	Wound	ERS13588132
975	FRC0725		806	533	322	SL533#	193	C. diphtheriae	Mitis	Negative		2019	Noumea	New Caledonia	Oceania	New Caledonia	Not documented	Cutaneous	Wound	ERS13588147
1001	FRC0751		828	533	322	SL533#	193	C. diphtheriae	Mitis	Negative		2019	Noumea	New Caledonia	Oceania	New Caledonia	Not documented	Cutaneous	Wound	ERS13588153
439	FRC0337		419	595	283	SL595#	172	C. diphtheriae	Gravis	Negative		2015	Le Mans	France	Europe	Mainland France	Not documented	Cutaneous	Not documented	ERS4330915
1011	FRC0762		836	595	514	SL595#	172	C. diphtheriae	Mitis	Negative		2019		Vanuatu	Oceania		Not documented	Throat	Throat	ERS13588163
80	SRR6816573	CD6	74	605	61	SL605	48	C. diphtheriae	Mitis			2012		Australia	Oceania		Not documented	Cutaneous (leg)		PRJNA436425
86	SRR6816580	CD16	80	605	61	SL605	48	C. diphtheriae	Mitis			2014		Australia	Oceania		Not documented	Cutaneous (unknown site)		PRJNA436425
88	SRR6816581	CD17	82	605	61	SL605	48	C. diphtheriae	Mitis			2014		Australia	Oceania		Not documented	Cutaneous (unknown site)		PRJNA436425
863	FRC0579		716	605	61	SL605	48	C. diphtheriae	Mitis	Negative		2018	Noumea	New Caledonia	Oceania	New Caledonia	None	Cutaneous	Wound	ERS13588129
71	SRR6816561	CD46	65	687	52	SL687	42	C. diphtheriae	Mitis			2016		Australia	Oceania		Not documented	Cutaneous		PRJNA436425

927	SRR6816604	CD30	771	687	485	SL687	42	C. diphtheriae	Mitis			2015		Australia	Oceania		Not documented	Cutaneous (unknown site)		PRJNA436425
998	FRC0748		825	687	510	SL687	42	C. diphtheriae	Belfanti	Negative		2019	Noumea	New Caledonia	Oceania	New Caledonia	Not documented	Not documented	Central venous catheter	ERS13588150
76	SRR6816567	CD31	70	744	57	SL744	45	C. diphtheriae	Mitis			2015		Australia	Oceania		Not documented	Cutaneous (unknown site)		PRJNA436425
517	FRC0515		487	524	57	SL744	45	C. diphtheriae	Mitis	Negative		2017	Noumea	New Caledonia	Oceania	New Caledonia	Not documented	Cutaneous	Not documented	ERS4330976
536	FRC0547		505	524	57	SL744	45	C. diphtheriae	Mitis	Negative		2017	Noumea	New Caledonia	Oceania	New Caledonia	Not documented	Cutaneous	Wound	ERS4330989
865	FRC0581		718	524	57	SL744	45	C. diphtheriae	Mitis	Negative		2018	Noumea	New Caledonia	Oceania	New Caledonia	None	Cutaneous	Abscess	ERS13588131

alias defined in this study
